# Decoding the Chloroplast Genome of *Tetrastigma* (Vitaceae): Variations and Phylogenetic Selection Insights

**DOI:** 10.3390/ijms25158290

**Published:** 2024-07-29

**Authors:** Junqiao Zhu, Yang Huang, Weiguo Chai, Pengguo Xia

**Affiliations:** 1 College of Life Sciences and Medicine, Zhejiang Sci-Tech University, Hangzhou 310018, China; 2 Institute of Biotechnology, Hangzhou Academy of Agricultural Sciences, Hangzhou 310024, China; kuni@21cn.com

**Keywords:** chloroplast genome, *Tetrastigma*, comparative genomics, phylogenetic analysis, selective pressure analysis

## Abstract

*Tetrastigma* (Vitaceae) is known for its ornamental, medicinal, and ecological significance. However, the structural and variational characteristics of the *Tetrastigma* chloroplast genome and their impact on phylogenetic relationships remain underexplored. This study utilized bioinformatics methods to assemble and annotate the chloroplast genomes of 10 *Tetrastigma* species and compare them with five previously sequenced species. This study analyzed gene composition, simple sequence repeats, and codon usage patterns, revealing a high A/T content, uniquely identified pentanucleotide repeats in five species and several preferred codons. In addition, comparative analyses were conducted of the chloroplast genomes of 15 *Tetrastigma* species, examining their structural differences and identifying polymorphic hotspots (*rps16*, *rps16-trnQ*, *trnS*, *trnD*, *psbC-trnS-psbZ*, *accD-psaI*, *psbE-petL-petG*, etc.) suitable for DNA marker development. Furthermore, phylogenetic and selective pressure analyses were performed based on the chloroplast genomes of these 15 *Tetrastigma* species, validating and elucidating intra-genus relationships within *Tetrastigma*. Futhermore, several genes under positive selection, such as *atpF* and *accD*, were identified, shedding light on the adaptive evolution of *Tetrastigma*. Utilizing 40 Vitaceae species, the divergence time of *Tetrastigma* was estimated, clarifying the evolutionary relationships within *Tetrastigma* relative to other genera. The analysis revealed diverse divergences of *Tetrastigma* in the Miocene and Pliocene, with possible ancient divergence events before the Eocene. Furthermore, family-level selective pressure analysis identified key features distinguishing *Tetrastigma* from other genera, showing a higher degree of purifying selection. This research enriches the chloroplast genome data for *Tetrastigma* and offers new insights into species identification, phylogenetic analysis, and adaptive evolution, enhancing our understanding of the genetic diversity and evolutionary history of these species.

## 1. Introduction

The genus *Tetrastigma*, belonging to the Vitaceae family, comprises numerous climbing plants predominantly distributed in tropical and subtropical regions, especially in Asia. These plants are favored by horticulturists for their ornamental foliage and climbing ability. Due to their unique tuberous root structures, some *Tetrastigma* species are rich in starch and possess edible value [1]. Additionally, *Tetrastigma* species hold significant medicinal value, with notable antioxidant and anti-inflammatory properties [2,3], and numerous studies have demonstrated their anti-tumor effects [4,5].

**Figure 1:**
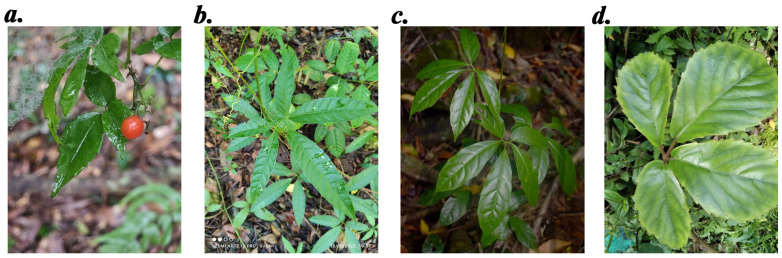
Four species of *Tetrastigma*, from left to right: (**a**) *Tetrastigma hemsleyanum*. (**b**) *Tetrastigma leucostaphylum*. (**c**) *Tetrastigma thorsborneorum*. (**d**) *Tetrastigma voinierianum*.

Despite their distinct morphological traits (Figure 1), the classification and identification of *Tetrastigma* species are challenging due to the considerable number of species, considerable morphological variation, and the occurrence of hybridization. Traditional morphological characteristics can be influenced by environmental and growth conditions, further complicating classification and identification.

Common methods for phylogenetic identification of species include ITS sequences and SCoT molecular markers [6]. Simple sequence repeats (SSRs) are also used as molecular markers. SSRs, also known as microsatellites, consist of tandem repeats of nucleotide sequences, such as mononucleotides and dinucleotides. Researchers have reported the successful application of SSR markers in maize hybrid genotypes and disease-resistant tomato varieties [7,8], indicating the feasibility of SSRs as molecular markers. Single nucleotide polymorphism (SNP) markers also offer great advantages. SNPs are highly specific, as they represent variations at a single nucleotide such as nucleotide transversions with each SNP representing a nucleotide difference [9]. Boakyewaa et al. analyzed 94 early-maturing maize yellow and white varieties based on SNP markers and morphological traits, successfully identifying distinct clusters of inbred lines [10]. Exploring SSRs and SNPs in *Tetrastigma* species holds significant value.

Chloroplast is a crucial organelle in plant cells, with a relatively conserved genome structure and unique features such as the *ycf1* DNA barcode. Research on chloroplast genomes offers several advantages: (1) The chloroplast genome is relatively small and simple in structure, making it easy to assemble and analyze without consuming extensive computational resources. Additionally, the chloroplast genome has a highly conserved quadripartite circular structure, divided into a large single-copy region (LSC), a small single-copy region (SSC), and a pair of inverted repeats (IRs) [11]. By comparing the gene boundary distances between different species’ chloroplast genomes, researchers can analyze phenomena such as rearrangements. (2) The chloroplast genome, though small, contains abundant genetic information, aiding in phylogenetic and species identification studies, for example, SSRs and SNPs mentioned earlier. (3) The evolutionary stability of the chloroplast genome makes it suitable for interspecies and intraspecies phylogenetic analysis. Researchers have reported that the evolutionary rate of synonymous substitution in the chloroplast genome is half that of the nuclear genome [12], indicating the relative stability and conservativeness of the chloroplast genome.

Furthermore, the simple structure and relatively small size of the chloroplast genome make more extensive horizontal research possible, such as pan-chloroplast genomes. Distinct from conventional chloroplast genome comparative analysis, research on pan-chloroplast genomes typically involves dozens to hundreds of samples, which can be different species or different varieties of the same species. The methods of pan-chloroplast genome research have been applied, for example, to help develop molecular markers for peppers and clarify genetic variation relationships [13]. In addition, research on genetic variation and temperature adaptation in cucumbers [14], as well as research on accession-specific markers in the genus *Hibiscus* [15], have all been supplemented by the methods of pan-chloroplast genome research. Whether it is pan-chloroplast genomes or conventional chloroplast genome comparative analysis, both can deepen people’s understanding of molecular markers, phylogenetics, and other aspects.

With the advancement of next-generation sequencing technologies and the reduction in sequencing costs, chloroplast genomes have been widely used in phylogenetic analyses in recent years [16,17,18], demonstrating the feasibility of using chloroplast genomes for phylogenetic analysis and species identification. Recent research has reported that nearly 13,000 plant chloroplast genomes have been published (NCBI, September 2023) [12]. In recent years, the complete chloroplast genomes of species such as *Tetrastigma hemsleyanum* and *Tetrastigma planicaule* have been assembled and annotated [19,20]. With ongoing sequencing studies and improvements in assembly software [21], more data resources for *Tetrastigma* species are being supplemented. However, data alone are not enough; corresponding analyses must follow. Compared to other genera in the Vitaceae family, evolutionary relationship studies on *Tetrastigma* are still limited. Although some studies have analyzed the phylogenetic relationships of *Tetrastigma* based on ten chloroplast DNA regions [22], they only include a portion of the *Tetrastigma* species. Some *Tetrastigma* species only have sequencing data with limited related studies, especially on chloroplast genomes.

When studying phylogenetic relationships, constructing phylogenetic trees based on methods such as Maximum Likelihood (ML) and maximum parsimony (MP) is an effective approach. Additionally, a time-calibrated tree allows for researchers to delve into species’ evolutionary relationships over long time scales, clarifying some controversies arising from constructing phylogenetic trees using conventional methods. Nie and colleagues reconstructed the phylogenetic relationships of the genus *Ampelopsis* within the Vitaceae family, providing detailed divergence times using fossil data combined with global geography [23]. Similarly, the evolutionary time scale of the genus *Vitis* has also been studied [24]. Selecting an appropriate molecular clock is crucial in molecular dating studies. Previous research has analyzed the divergence times and absolute substitution rates of six genera: *Ampelocissus*, *Ampelopsis*, *Nekemias*, *Parthenocissus*, *Rhoicissus*, and *Vitis* under different molecular clocks [24]. However, studies on the molecular clock selection and time scale for the genus *Tetrastigma* are still lacking, or they are merely used as reference outgroups.

Selective pressure analysis is an important method for studying the evolutionary processes of species. By analyzing selective pressure, researchers can understand the selective pressure on genes or genomes during evolution, revealing the conservation and adaptive changes in gene functions. Nonsynonymous substitution rates (*Ka*) and synonymous substitution rates (*Ks*) are important parameters for measuring gene evolutionary rates. *Ka* represents the rate of mutations that lead to amino acid changes in protein-coding genes, while *Ks* represents the rate of mutations that do not change amino acids. The *Ka/Ks* ratio is a crucial indicator for assessing gene selective pressure. Researchers have used the *Ka/Ks* ratio to explore the selective pressure on *Lilium ledebourii* [25]. During the evolution of *Tetrastigma* species, what selective pressure was applied by the key genes distinguishing this genus from others experience? How did evolution occur within the genus? These questions are worth in-depth research.

Our study aims to address the following questions: (1) What are the SSR and SNPs molecular markers for species identification in the *Tetrastigma* genus? (2) Based on chloroplast genome data, what are the phylogenetic relationships within the *Tetrastigma* genus? (3) What is the divergence time of *Tetrastigma* species, and how does it compare to the evolutionary timeline of other genera within the Vitaceae family? (4) What selective pressures have key genes within the *Tetrastigma* genus experienced during evolution, and how do these pressures compare to those in other genera within the Vitaceae family? Thus, we selected ten sequenced species: *Tetrastigma thorsborneorum*, *Tetrastigma serrulatum*, *Tetrastigma pyriforme*, *Tetrastigma pachyphyllum*, *Tetrastigma nilagiricum*, *Tetrastigma leucostaphylum*, *Tetrastigma cauliflorum*, *Tetrastigma canarense*, *Tetrastigma annamense*, and *Tetrastigma angustifolium* and assembled and annotated their respective chloroplast genomes. We then explored the characteristics of each genome and compared them with the genomes of five other *Tetrastigma* species. Our analysis included codon usage patterns, simple sequence repeats (SSRs), and hotspot region identification, aiming to screen for promising identification sites. Additionally, as the phylogenetic relationships within *Tetrastigma* are unclear, we constructed phylogenetic trees based on Maximum Likelihood and Bayesian methods for the genus *Tetrastigma* and the Vitaceae family, estimating divergence times to further supplement and verify the evolutionary position and phylogenetic relationships of *Tetrastigma*. Based on the phylogenetic trees, we also analyzed selective pressure within the genus *Tetrastigma* and among genera, aiming to clarify the roles of different genes in the evolution of *Tetrastigma* from both intra- and intergeneric perspectives.

## 2. Results

### 2.1. Genome Organization and Features

**Figure 2:**
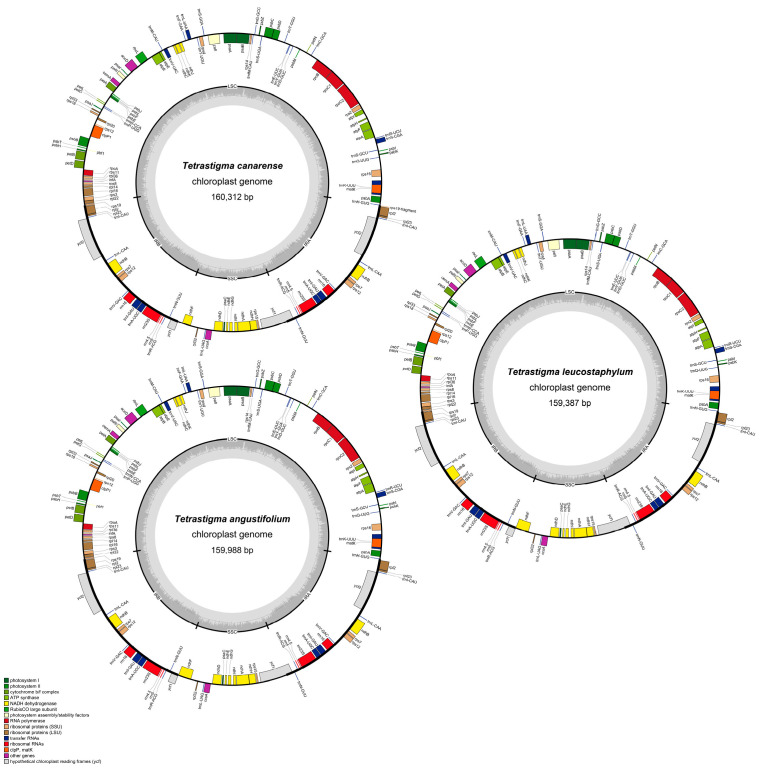
Chloroplast genome maps of *Tetrastigma*, taking *T. angustifolium*, *T. canarense* and *T. leucostaphylum* as examples.

The chloroplast genomes of ten *Tetrastigma* species were successfully assembled, resulting in gap-free, circularly structured genomes (Figure 2). Additionally, chloroplast genomes of five other *Tetrastigma* species were downloaded from NCBI, allowing for the analysis of a total of fifteen species. The sizes of these 15 *Tetrastigma* chloroplast genomes (Table 1) range from 159,387 base pairs (bp) in *T. nilagiricum* to 160,736 bp in *T. annamense*, with total GC content varying between 37.35% in *T. voinierianum* to 37.62% in *T. cauliflorum*. All genomes exhibit the typical quadripartite structure, consisting of a large single-copy (LSC) region, a pair of inverted repeats (IRs), and a small single-copy (SSC) region. The LSC regions range in length from 87,499 bp in *T. nilagiricum* to 88,821 bp in *T. annamense*, the IR regions range from 26,288 bp in *T. annamense* to 26,934 bp in *T. thorsborneorum*, and the SSC regions range from 18,649 bp in *T. thorsborneorum* to 19,339 bp in *T. annamense*. The corresponding GC contents for these regions are 35.34–35.63%, 42.73–42.99%, and 31.41–31.79%, respectively.

Excluding the pseudogenes *ψycf1* and *ψrps19-fragment*, the *Tetrastigma* chloroplast genomes encode a total of 130 genes, comprising 85 CDS, 8 rRNA genes, and 37 tRNA genes. These genes are broadly categorized into four types (Table 2): self-replication genes, photosynthesis-related genes, other genes, and genes of unknown function, with the majority located in the LSC region.

### 2.2. SSRs Analysis

Overall, 1139 SSRs were detected. *T. lawsonii* and *T. cauliflorum* have the highest number of SSRs, totaling eighty-three, while *T. thorsborneorum* has the fewest, with only sixty-four. Mapping the identified simple sequence repeats to the chloroplast genome reveals the distribution regions of SSRs. The results indicate that SSRs in the fifteen species are primarily distributed in the LSC region, followed by the SSC region, with the least in the IR regions, where the numbers of SSRs detected in IRa and IRb are equal (Table 3).

**Figure 3:**
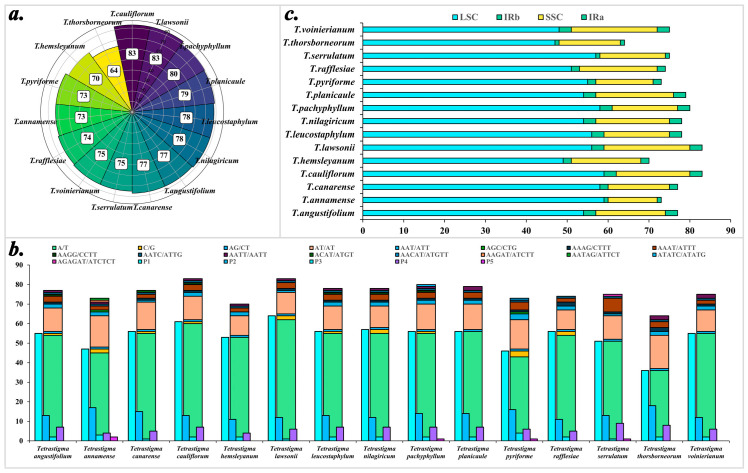
Analysis of chloroplast genome SSRs in 15 *Tetrastigma* species. (**a**) The total number of SSRs in the 15 species; (**b**) The total number of repeat types classified by repeat unit type and repeat unit count. P1—mono, P2—di, P3—tri, P4—tetra, P5—penta; (**c**) Statistics of SSR distribution region.

From the perspective of nucleotide repeat count (Figure 3), the SSRs in the 15 *Tetrastigma* species mainly consist of mononucleotide repeats (70.68%), followed by dinucleotide repeats (17.91%), trinucleotide repeats (2.63%), tetranucleotide repeats (8.34%), and pentanucleotide repeats (0.44%). Among them, pentanucleotide repeats are very rare. In terms of nucleotide composition types (Table S2), considering sequence complementarity, a total of seventeen repeat types were detected, mainly A/T and AT/AT. A detailed analysis of different nucleotide composition types, considering only mononucleotide and dinucleotide repeats, shows that repeats containing C and G bases are exceedingly rare, with A/T and AT/AT accounting for 69.27% and 16.59%, respectively, while C/G and AG/CT account for only 1.40% and 1.32%. This trend is also observed in tetranucleotide repeats, where the number of SSRs without C/G is greater than those with C/G, except for the pairs AAAG/CTTT and AATC/ATTG. However, the total number of these repeats is too small to reliably analyze frequencies solely based on count.

Notably, pentanucleotide repeats were detected in 4 of the 15 *Tetrastigma* species: *T. annamense*, *T. pachyphyllum*, *T. pyriforme*, and *T. serrulatum*.

### 2.3. Codon Usage Bias Analysis

Codon usage and amino acid frequency were analyzed for the fifteen species, excluding stop codons. The number of codons used across the fifteen species showed insignificant variation, ranging from 49,986 in *T. leucostaphylum* to 50,614 in *T. pyriforme* with an average of 50,300 codons. The twenty amino acids are encoded by sixty-one codons, with Leu, Arg, Ser, and Phe being the most common, and Met and Trp being the least common. Among the sixty-one codons, TTT and AAA are the most frequent, while CGC and GCG are the least frequent.

**Figure 4:**
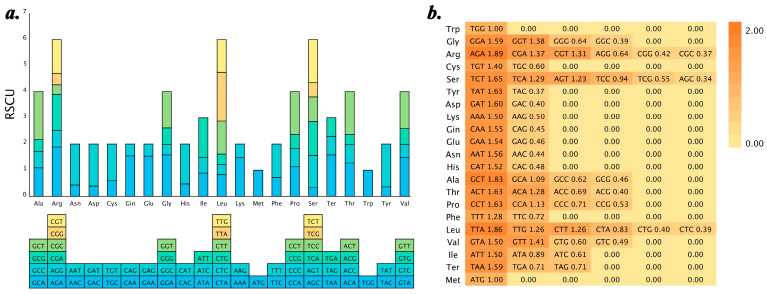
Relative synonymous codon usage (RSCU) in *Tetrastigma* plants, taking *T. canarense* as an example. (**a**) Stacked bar chart of the relative synonymous codon usage (RSCU) in *Tetrastigma* plants; (**b**) Heatmap displaying RSCU values.

RSCU values were calculated, excluding RNA editing and only considering genes with the start codon ATG (Figure 4). The usage frequency of codons was determined by an RSCU value of one, with RSCU > 1 indicating higher usage frequency. For the sixteen species analyzed, RSCU results were presented in the form of stacked bar charts and heatmaps, where the color intensity in the heatmaps represented the magnitude of the RSCU values. The results showed that 30 codons had RSCU > 1, while Trp and Met both had RSCU values equal to one. Most codons tend to end with A/T, with a few exceptions such as Leu (TTG) and Ser (TCG), which end with G.

### 2.4. Comparative Chloroplast Genome Analysis

**Figure 5:**
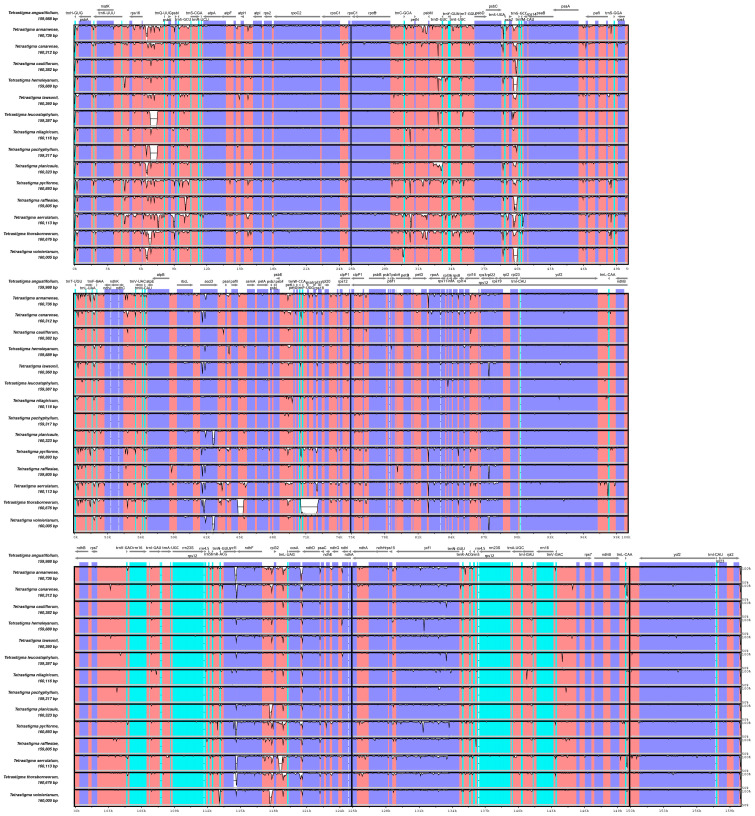
Visualization of chloroplast genome alignment of 14 *Tetrastigma* species, using *T. angustifolium* as a reference. The vertical scale represents the percentage of identity, ranging from 50% to 100%. The horizontal axis denotes the coordinates within the chloroplast genome. Colors are used to indicate distinctive features.

Using the chloroplast genome sequence of *T. angustifolium* as a reference, the mVISTA program was employed to conduct a comparative analysis of the chloroplast genomes of the remaining 14 *Tetrastigma* species (Figure 5). The results indicated that the 15 chloroplast genomes exhibit minor differences, with coding region sequences showing less variation than non-coding regions, except for genes such as *ycf1* and *accD*. Significant differences were found in regions such as *rps16-trnR^UCU^*, *trnC^GCA^-trnT^GGU^*, *trnS^UGA^-trnG^GCC^*, and *ndhF-trnL^UAG^*.

**Figure 6:**
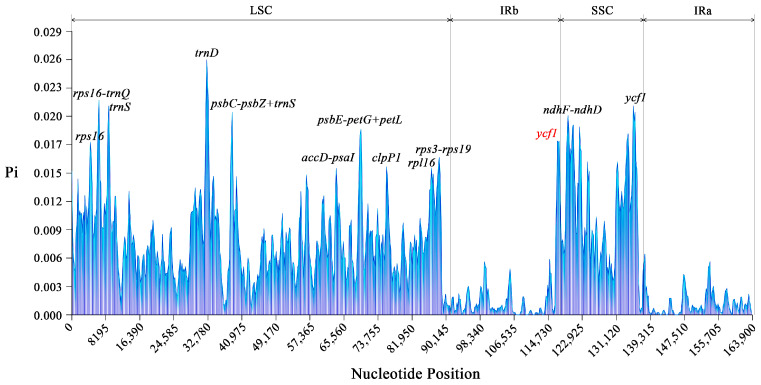
Nucleotide diversity analysis based on sliding windows and the *Tetrastigma* chloroplast genome sequences. The *X*-axis represents the length coordinates of the multiple sequence alignment of the fifteen species. The *Y*-axis represents the nucleotide diversity at each position corresponding to each window. Red represents pseudogenes.

Nucleotide polymorphism (Pi) analysis of the chloroplast genomes of the 15 *Tetrastigma* species was performed using DnaSP V6. The results (Figure 6) showed that the Pi values of the 15 chloroplast genomes ranged from 0 to 0.02614, with an average Pi value of 0.006013. A total of 13 nucleotide polymorphism hotspots with Pi > 0.015 were detected, with 10 found in the LSC region: *rps16*, the intergenic region between *rps16* and *trnQ*, *trnS*, *trnD*, the intergenic region between *psbC-trnS-psbZ*, the intergenic region between *accD-psaI*, *psbE-petL-petG*, *clpPI*, *rpl16*, and *rps3-rps19*. Two hotspots were detected in the SSC region: *ndhF-ndhD* and *ycfI*. One hotspot was detected in the IR region, which is a pseudogene fragment of *ycf1*. The number of polymorphic sites in the LSC and SSC regions was greater than in the IR region.

### 2.5. IR/SC Boundaries Analysis

**Figure 7:**
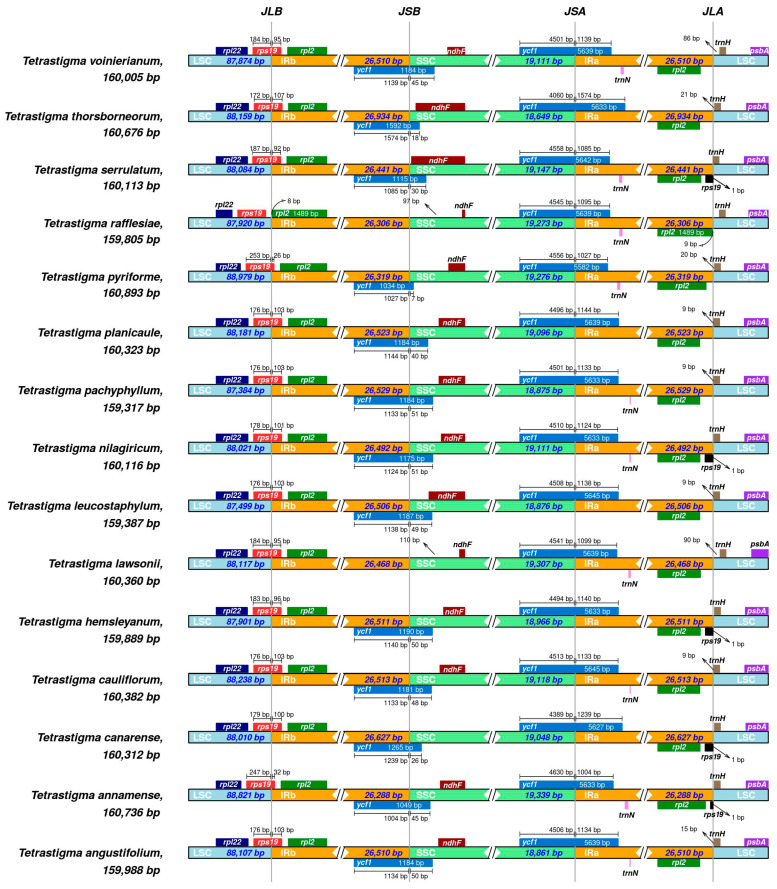
Comparison of LSC, SSC, and IR boundaries in the chloroplast genomes of 15 *Tetrastigma* species. Genes are annotated in assorted colors and are labeled with their distances from the boundaries and the lengths of these distances. The lengths of these color blocks are not indicative of the gene lengths but are compressed or expanded based on the differing distances. LSC, SSC, and IR regions are represented in assorted colors. JLB, JSB, JSA, and JLA, respectively, denote the boundaries of the four regions.

IRScope was used to compare the IR/SC boundary regions of the 15 *Tetrastigma* species. (Figure 7). The results indicate that *psbA* was consistently located in the LSC region, at the IRa/LSC boundary. The distance from *trnH* to the boundary varied from 1 to 94 bp, all within the LSC region, specifically the distances for *T. planicaule*, *T. pachyphyllum*, *T. leucostaphylum*, and *T. cauliflorum* were all 9 bp, whereas the distances for *T. lawsonii* and *T. voinierianum* were 90 bp and 86 bp, which is significantly different from the other species. The *rpl22* gene was consistently located in the LSC region, while the *rps19* gene, except for *T. rafflesiae*, spanned the LSC/IRb boundary in the other fourteen species, with the portion in IRb ranging from 26 to 107 bp from the LSC/IRb boundary. The *ndhF* gene was in the SSC region, with distances from the IRb/SSC boundary ranging from 4 to 110 bp. The *ycf1* gene (5582–5645 bp) spanned the SSC/IRa boundary, and genes partially spanning IR and SC regions were often annotated as pseudogenes, such as the *ycf1* pseudogene at the IRb and SSC boundary. Similarly, in the 15 species, certain species such as *T. serrulatum* had fragments of the *rps19* gene annotated in the IRa region, which were also considered pseudogene fragments without the original gene function.

### 2.6. Phylogenetic Analysis

**Figure 8:**
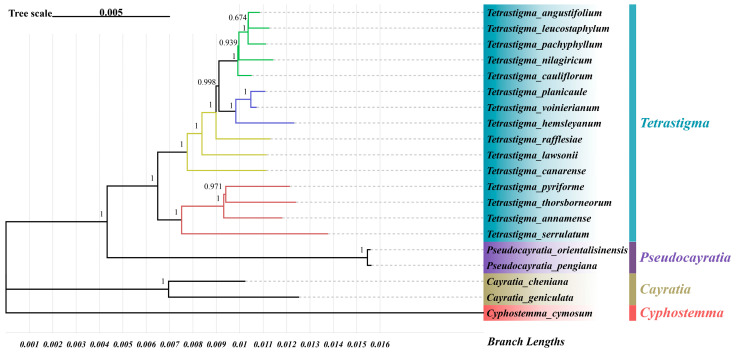
Maximum Likelihood phylogenetic tree based on complete chloroplast genomes, with *Pseudocayratia pengiana*, *Pseudocayratia orientalisinensis*, *Cayratia geniculate*, *Cayratia cheniana*, and *Cyphostemma cymosum* as outgroups. Bootstrap values are displayed on the branches.

A phylogenetic tree was constructed based on the complete chloroplast genome sequences of the 15 *Tetrastigma* species, using species from the genera *Pseudocayratia* (*Pseudocayratia pengiana*, *Pseudocayratia orientalisinensis*), *Cayratia* (*Cayratia geniculate*, *Cayratia cheniana*), and *Cyphostemma* (*Cyphostemma cymosum*) as outgroups. The results (Figure 8) indicated that all four genera originated from the same clade. The *Tetrastigma* species clustered into a monophyletic group with 100% bootstrap support, indicating high confidence in the results. Within *Tetrastigma*, *T. serrulatum*, *T. annamense*, *T. thorsborneorum*, and *T. pyriforme* formed one clade, while the remaining species formed another clade, both with 100% bootstrap support. Additionally, *T. voinierianm* and *T. planicaule* formed a clade with *T. hemsleyanum*, and *T. leucostaphylum* and *T. angustifolium* formed a clade with *T. cauliflorum*, *T. nilagiricum*, and *T. pachyphyllum*.

### 2.7. Divergence Time Estimation

**Figure 9:**
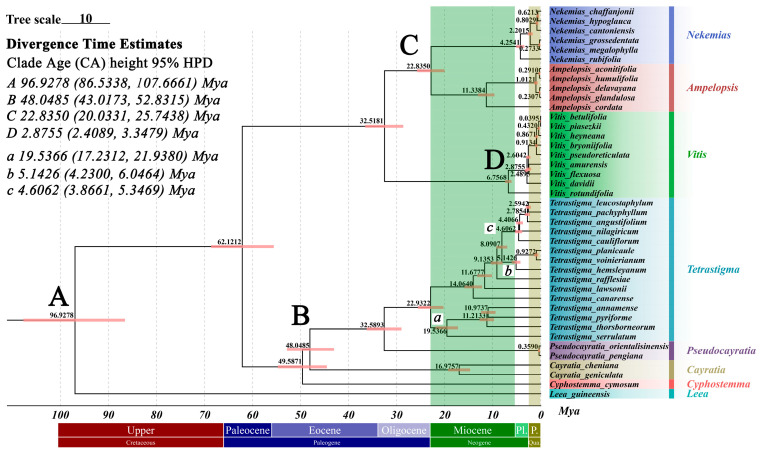
Divergence times of Vitaceae and *Tetrastigma* species estimated based on the shared CDS sequences of the chloroplast genome, with *Leea guineensis* from the Leeaceae as the outgroup. The mean divergence times are shown next to the nodes, and the red bars correspond to the Clade Age height 95% HPD. (**A**–**D**) represent clade nodes used for priors, where the values indicate the results of estimated divergence times in this analysis, not priors. Specifically, (**A**) represents the deep calibration point for *Leea* and Vitaceae; (**B**) the existing fossil record of *Tetrastigma*; (**C**) for the genus *Ampelopsis*; (**D**) for the subgenus *Vitis*. (**a**–**c**) indicate the estimated divergence times within *Tetrastigma*, corresponding to the results in 2.6. The bottom of the figure shows the Geologic Time Scale, with the Lower Epoch of the Cretaceous Period (145–100.5 Mya) and the Holocene Epoch of the Quaternary Period (0.0117 Mya–present) omitted.

The Bayesian phylogenetic tree (Figure 9) constructed for 41 species within the Vitaceae family reveals detailed divergence times and significant evolutionary events within the family. This tree includes representatives from different genera: *Nekemias*, *Ampelopsis*, *Vitis*, *Tetrastigma*, *Pseudocayratia*, *Cayratia*, and *Cyphostemma*, with an outgroup reference from the Leeaceae family, *Leea*. The phylogenetic tree is time-calibrated, with the x-axis marking geological periods (Cretaceous, Paleogene, and Neogene). Nodes A-D represent clade nodes used for priors, where the values indicate the estimated divergence times in this analysis, not the actual priors. The outgroup, *Leea guineensis*, diverged from the Vitaceae family in the Late Cretaceous, around 96.93 Mya (95% HPD: 86.53–107.67 Mya), indicating a distant relationship and supporting its use as an outgroup. The phylogenetic tree shows clear genus-level separations. At 62.12 Mya, the Vitaceae family split into two branches, with *Tetrastigma* located in the Cayratieae subfamily. Within the Cayratieae subfamily, *Pseudocayratia* diverged from *Tetrastigma* around 32.59 Mya, closely following the divergence from *Vitis*. The divergence times for *Cayratia* and *Cyphostemma* suggest an older history, dating back to 49.59 Mya. For *Tetrastigma*, the topology of the Bayesian tree is largely consistent with that constructed using the Maximum Likelihood (ML) method, with minor differences. These differences appear mainly at nodes with low bootstrap values in the ML tree, such as the relationships among *T. angustifolium*, *T. leucostaphylum*, and *T. pachyphyllum*. In the Bayesian tree, *T. leucostaphylum* and *T. pachyphyllum* show a closer relationship, unlike in the ML tree where *T. angustifolium* is closer. Following the ML method results, we artificially divided the evolution of *Tetrastigma* into three clades: Subclade a: diverged around 19.54 Mya (95% HPD: 17.23–21.94 Mya) in the Miocene; Subclade b: diverged around 5.14 Mya (95% HPD: 4.23–6.05 Mya) in the Pliocene; Subclade c: diverged around 4.61 Mya (95% HPD: 3.87–5.35 Mya) in the Pliocene. Diverse divergences occurred in the Miocene, with recent diversification in the Pliocene. Overall, most *Tetrastigma* species in this study diverged before the Pleistocene, earlier than most of the selected *Vitis* species.

### 2.8. Selective Pressure Analyses

**Figure 10:**
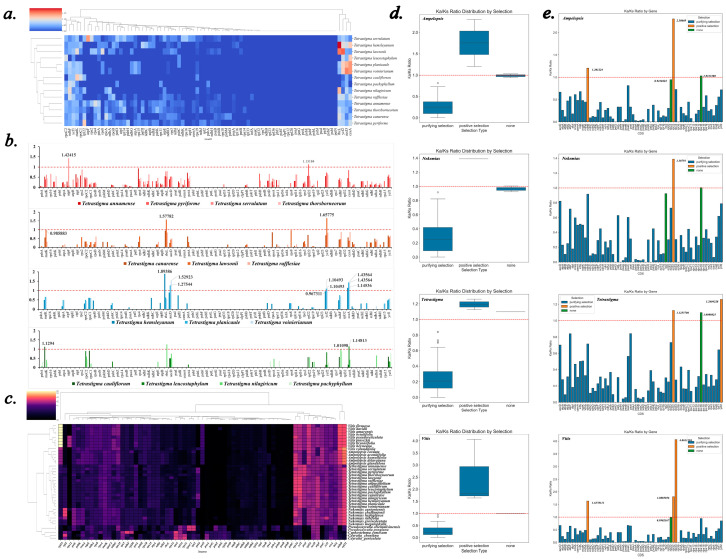
Selective pressure analysis results. (**a**) Cluster heatmap showing the *Ka/Ks* values of chloroplast genomes from 14 species, using *Tetrastigma angustifolium* as a reference, the *Ka/Ks* value varies between 0 and 2, corresponding to a color range of blue to red. (**b**) A set of graphs displaying the *Ka/Ks* values of each gene, dividing 14 species into four groups according to the result of the phylogenetic tree. (**c**) Cluster heatmap showing the *Ka/Ks* values of chloroplast genomes from 40 species, using *Leea guineensis* as a reference. The *Ka/Ks* value varies between 0 and 5, corresponding to a color range of black to yellow. (**d**) Box plots divided into four units by genus, taking the arithmetic mean of the *Ka/Ks* values of the common CDS of different species and obtaining a box plot to display the distribution of data. (**e**) Bar charts drawn by dividing the *Ka/Ks* values of the common CDS of different species into four units based on their genus, taking the arithmetic mean. Orange represents positive selection, blue represents purifying selection, and green represents *Ka/Ks* close to 1, indicating no significant selection.

The *Ka/Ks* ratio was used to distinguish the types of selective pressures acting on genes, with *Tetrastigma angustifolium* as the reference. *Ka* and *Ks* values were calculated for the other 14 *Tetrastigma* species (Figure 10a). Based on the phylogenetic tree, selective pressure analysis was conducted by grouping the species accordingly (Figure 10b). The results indicated that some genes in *Tetrastigma* species are under positive selection, while most are under purifying selection.

In the clade consisting of *T. serrulatum*, *T. annamense*, *T. thorsborneorum*, and *T. pyriforme*, only the *T. serrulatum*’s *atpF* gene had a *Ka/Ks* ratio of 1.42415, indicating positive selection. Similarly, *T. pyriforme*’s *rpl22* gene had a *Ka/Ks* ratio of 1.13184, also under positive selection. In contrast, the *atpF* and *rpl22* genes in the remaining twelve species had *Ka/Ks* ratios of less than one, indicating purifying selection. Additionally, most genes across these species were under purifying selection. The remaining three clades (*T. canarense*, *T. lawsonii*, *T. rafflesiae*; * T. voinierianm*, *T. planicaule*, *T. hemsleyanum*; * T. leucostaphylum*, *T. cauliflorum*, *T. nilagiricum*, *T. pachyphyllum*) showed some common patterns. In *T. lawsonii* and *T. hemsleyanum*, the *rbcL* gene had *Ka/Ks* ratios of 1.57782 and 1.29386, respectively, indicating positive selection. The *ccsA* gene in *T. voinierianm*, *T. planicaule*, and *T. hemsleyanum* had *Ka/Ks* ratios greater than one, also suggesting positive selection.

Additionally, an analysis was conducted on 40 species of the Vitaceae family, using *Leea guineensis* from the Leeaceae family as a reference species. *Ka/Ks* ratios for 70 CDS sequences from these 40 species were calculated, with genes having a high proportion of NA values filtered out, resulting in 66 CDS being analyzed. A cluster heatmap displayed the specific *Ka/Ks* values for species and genes (Figure 10c), showing that most genes were dark-colored, indicating significant purifying selection. Furthermore, box plots (Figure 10d) and bar charts (Figure 10e) were used to illustrate the distribution and specific values, as well as the type of selection.

Most Vitaceae species, except those from the *Pseudocayratia*, *Cayratia*, and *Cyphostemma*, exhibited significant bright areas in the *rps16*-*rpl32* region, suggesting strong positive selective pressures in this region, particularly on *rpl32*. In *Ampelopsis* species, *rpl32* and *ndhB* showed significant positive selection, with arithmetic mean *Ka/Ks* ratios of 2.307 and 1.201, respectively. Although the *Ka/Ks* ratio for the *rps16* gene was greater than one, it was not significant, similar to the *rpl22* gene, indicating that neither *rps16* nor *rpl22* underwent significant purifying or positive selection. In the Nekemias species, the *Ka/Ks* ratio for *rpl32* was 1.388, indicating significant positive selection, while the *Ka/Ks* ratios for *rps16* and *rpl2* were not significantly different from 1. In the *Tetrastigma* species, both *rpl32* and *ycf2* showed significant positive selection, with arithmetic mean *Ka/Ks* ratios of 1.126 and 1.260, respectively. As in the other genera, the *rps16* gene did not have a significantly higher *Ka/Ks* ratio than one. In the *Vitis* species, *rpl33*, *rpl32*, and *ndhB* were under significant positive selection, with arithmetic mean *Ka/Ks* ratios of 4.061, 1.809, and 1.638, respectively. The *rpl22* gene, however, was not under significant positive or purifying selection after significance analysis. Genes under significant positive selection in the four genera are recorded (Table 4), along with data on normality and the tests used. Significance analysis was conducted in groups due to considerable inter-genera differences for some genes. The *Pseudocayratia*, *Cayratia*, and *Cyphostemma* exhibited more differentiated characteristics, particularly in the *rps16*-*rpl32* region, which was inconsistent with other genera. In these genera, genes such as *rps16*, *psaI*, *psaC*, *ndhB*, and *rpl32* might be under purifying selection. However, due to the small number of species, no significance analysis was performed for these genera.

## 3. Discussion

### 3.1. Chloroplast Genome Basic Characteristics

*Tetrastigma* is renowned for its ornamental and medicinal significance, and there is a substantial market demand for it. Although the *Tetrastigma* genus encompasses a variety of species, most of research to date has focused on *Tetrastigma hemsleyanum*, which has limited resources. The breeding and development foundation of *Tetrastigma* species is weak. Previous studies on chloroplast genomic DNA regions (*atp B-rbcL*, *atp F-atpH*, *matK*, *psbK-psbI*, *rbcL*, *rpoC1*, *rps16*, *trn C-petN*, *trn H-psbA*, *trn L-trnF*) and the seed and plant morphological data of the *Tetrastigma* species [22] have laid a theoretical groundwork for the development of *Tetrastigma*. This study supplements the aforementioned research by analyzing the genomic characteristics and genetic relationships of fifteen species within the genus based on complete chloroplast genome data. The primary methodology employed was bioinformatics analysis, which involved the assembly of genomes from ten species and the examination of the chloroplast genomes from fifteen species. The size of the *Tetrastigma* chloroplast genome ranges from 159,387 (*T. nilagiricum*) to 160,736 bp (*T. annamense*). In terms of GC content, the GC content in the IR regions is generally higher than that in the LSC and SSC regions, a pattern commonly observed across other species [16,26]. This distribution of GC content may be related to the replication mechanism and gene expression regulation of the chloroplast genome. Šmarda et al. have studied the GC content in monocotyledonous plants and suggested that GC-rich DNA might have an advantage during cellular freezing and desiccation processes, which may imply a potential impact of GC content on the stability of gene expression under specific environmental conditions [27], which may enhance the plants’ resistance to drought and low-temperature stress. The structure of the chloroplast genome, composed of the LSC region, a pair of IR regions, and the SSC region, forms the typical quadripartite structure. The length of the IR regions varies from 26,288 bp (*T. annamense*) to 26,934 bp (*T. thorsborneorum*), while the length of the SSC region ranges from 18,649 bp (*T. thorsborneorum*) to 19,339 bp (*T. annamense*). This quadripartite structure of the chloroplast genome is an adaptive feature formed during the long-term evolutionary process of plants, contributing to the stability, replication efficiency, and genetic diversity of the genome. Palmer et al. found that when large inverted repeat sequences are lost, chloroplast DNA undergoes more frequent rearrangements [28], and Perry et al. reported that the synonymous substitution rate of IR genes in leguminous plants is 2.3 times lower than that of single-copy (SC) genes [29]. The IR regions may play a role in preventing or reducing harmful effects during genome rearrangements. Additionally, apart from the pseudogenes *ψycf1* and *ψrps19-fragment*, the *Tetrastigma* chloroplast genome encodes a total of 130 genes, including 85 CDS, 8 rRNA genes, and 37 tRNA genes. The self-replicating genes are crucial for the replication and repair of chloroplast DNA, while the photosynthetic system genes are directly involved in the process of photosynthesis. Other genes and genes with unknown functions may play roles in the biosynthesis, energy conversion, and signal transduction processes within the chloroplast.

### 3.2. SSRs Analysis

SSRs are a common structural feature in the chloroplast genomes of plants. The results indicate that the predominant type of SSR is mononucleotide repeats, which are predominantly spatially located in the LSC region, with fewer other types of repeats, and the IR regions are highly conserved, mainly composed of A/T bp, similar to previous studies [30,31].

In both mononucleotide and dinucleotide repeats, the content of A/T (69.27%) and AT/AT (16.59%) is significantly higher than that of C/G (1.40%) and AG/CT (1.32%). This pattern is also observed in tetranucleotide repeats, where the number of SSRs without C/G is usually greater than those containing C/G, except for AAAG/CTTT and AATC/ATTG. However, looking at the total count, the number of trinucleotide repeats, pentanucleotide repeats, and similar types is too low to be representative, unlike mononucleotide and dinucleotide repeats.

The distribution and types of SSRs may vary among varied species due to a variety of factors, including genetic diversity, evolutionary pressure, and environmental adaptability. The number of repeats in SSRs can affect gene regulation, transcription, and protein function, providing a source of quantitative and qualitative variation [32]. Eukaryotic genomes contain a rich array of mononucleotide repeats (MNRs), and studies have shown that MNRs may have a certain relationship with single nucleotide polymorphisms (SNPs), possibly increasing the frequency of SNPs at adjacent positions [33]. The concentrated distribution of SSRs in the LSC region may be related to the higher gene density and functional diversity of this region, while the scarcity of SSRs in the IR regions may be related to their role in maintaining genomic stability. It is noteworthy that among the 15 *Tetrastigma* species, four species were detected to have pentanucleotide repeats: *T. annamense*, *T. pachyphyllum*, *T. pyriforme*, and *T. serrulatum*. These pentanucleotide repeats may play a role in the identification of these species, and in fact many studies have already used this approach for species identification [34], with related molecular markers also being developed. The diversity and distribution of SSRs are of significant importance for the adaptability and evolution of species and can serve as genetic markers to help study the genetic structure and intraspecific variation of species.

### 3.3. Condon Usage Analysis

This study conducted an in-depth analysis of codon usage and amino acid frequency across fifteen varied species, aiming to explore the conservation and variability in codon usage preferences among varied species. The results indicate that despite certain differences, there is a low variation in the overall number of codon usages among varied species, which may be closely related to the efficiency and accuracy of gene expression. During the evolutionary process, to maintain the stability of protein function, natural selection may favor the retention of codon usage patterns that can optimize the translation process. Most codons tend to end with A or T, which may be related to the stability of mRNA and translation efficiency. If the third position of a codon is A or T, it may be more easily recognized by tRNA, thereby increasing the translation speed. In fact, compared with other codons, the optimal codons rich in AT can generally reduce the energy required for mRNA folding, thus improving translation efficiency [35]. Our research results are consistent with this theory, and this preference may be universally present across varied species. The analysis of RSCU values further reveals the variability in codon usage frequency. We found that the RSCU values of thirty codons are greater than one, indicating that the usage frequency of these codons is higher than the average level.

### 3.4. IR/SC Boundaries Analysis

This study utilized IRScope to conduct an in-depth analysis of the IR/SC boundary regions of the chloroplast genomes in 15 *Tetrastigma* species. By comparing the gene distribution and variability at the IR/SC boundaries among varied species, we revealed the conservation and variability of the chloroplast genome structure, as well as the potential impacts of these variations on gene annotation and functional integrity. We found that the *rpl22* and *psbA* genes are located in the LSC region in all studied *Tetrastigma* species, further confirming the conservation of the LSC region in these species, consistent with the findings of Dong et al. [36]. In contrast, the *rps19* gene spans the LSC/IRb boundary in all but one species, *T. rafflesiae*, which is also in line with the results of Dong et al., who found that even within the same species the spatial situation of the chloroplast genome’s IR/SC boundary can vary, especially in the distribution of *rps19* and *rpl2* [36], indicating that there may be certain variability within the *Tetrastigma* genus. At the IRa/LSC boundary, the spatial length from the *trnH* gene to the boundary varies from 1 to 94 bp. Notably, the distances of *trnH* to the boundary in *T. lawsonii* and *T. voinierianum* are 90 and 86 bp, respectively, showing a significant difference from the other species, and this variability may indicate the uniqueness of these two species in their evolutionary process. Additionally, the position of the *ndhF* gene in the SSC region also exhibits some variability. The chloroplast genome is highly conserved, especially in the IR regions, and genes spanning the IR and SC regions are prone to having incomplete gene fragments annotated on the opposite side (usually considered pseudogenes), such as the segment of *ycf1* spanning SSC/IR that exists in IRa can be found in IRb, leading to the incorrect annotation of fragments of *ycf1*. This phenomenon is consistent with the results of Feng et al. [37,38].

### 3.5. Comparing the Polymorphism of Chloroplast Genomes

Based on the chloroplast genomes of 15 *Tetrastigma* species, the Pi value was calculated. In other species, such as those of the *Pleione* genus, 12 polymorphic sites were detected in the chloroplast genome at Pi > 0.011 [26]. However, even with a threshold set at Pi > 0.015, a considerable number of polymorphic sites (13 in total, some of which are formed by the connection of multiple genes) were still detected in the chloroplast genomes of *Tetrastigma* species. The results of mVISTA and Pi analysis together revealed polymorphic sites within the chloroplast genome such as *rps16*, trnS, and so on (*rps16-trnR^UCU^*, *trnC^GCA^-trnT^GGU^*, *trnS^UGA^-trnG^GCC^*, and *ndhF-trnL^UAG^*; 10 in LSC: *rps16*, *rps16-trnQ*, *trnS*, *trnD*, *psbC-trnS-psbZ*, *accD-psaI*, *psbE-petL-petG*, *clpPI*, *rpl16*, *rps3-rps19*; 2 in SSC: *ndhF-ndhD* and *ycfI*). Chloroplast markers developed based on polymorphic sites have a wide range of applications. Amar et al. used the *ycf1-ndhF* region as a plastid barcode for low-level taxonomic phylogenetic analysis of *Prunus persica*, validating the effectiveness of this site [39]. Therefore, based on the aforementioned detected polymorphic sites, corresponding chloroplast markers can be developed, which can accurately identify *Tetrastigma* species and are of great significance for their interspecific identification and phylogenetic analysis.

### 3.6. Phylogenetic Relationships and Divergence Time Estimation

#### 3.6.1. Phylogenetic Relationships

The *Tetrastigma* genus holds immense medicinal value, and precise identification of *Tetrastigma* species is crucial for the effective use of their medicinal resources. Therefore, understanding the phylogenetic relationships within the *Tetrastigma* genus has become a focal point of interest. This study, which constructed a phylogenetic tree based on chloroplast genome sequences, provides new insights into the evolutionary history and classification of *Tetrastigma* plants. The monophyletic clustering and high support values for *Tetrastigma* species underscore the stability of their taxonomy and their shared evolutionary lineage.

Previous studies have also attempted to elucidate the phylogenetic relationships within *Tetrastigma* using various molecular markers. For instance, Habib et al. constructed a phylogenetic tree for the *Vitaceae* family using 10 chloroplast DNA markers (*atpB-rbcL*, *atpF-atpH*, *matK*, *psbK-psbI*, *rbcL*, *rpoC1*, *rps16*, *trnC-petN*, *trnH-psbA*, *trnL-trnF*) and studied the phylogenetic relationships within the *Tetrastigma* genus [22]. This study corroborates some of the findings of that research, confirming the close phylogenetic relationship between *T. thorsborneorum* and *T. pyriforme*. Moreover, in the classification by Habib et al., *T. hemsleyanum*, *T. voinierianum*, and *T. planicaule*, although clustered into a larger group, still show some distance. Phylogenetic trees constructed using chloroplast DNA markers and those based on complete chloroplast genomes may differ, especially when the relationships are close. In fact, the analysis presented in this study suggests that, in addition to the chloroplast DNA markers used by Habib et al., genes such as *trnD* and *ycf1* are also suitable as markers [39,40]. Considering more factors comprehensively may lead to more refined results.

The clustering relationship of *T. cauliflorum*, *T. nilagiricum*, *T. pachyphyllum*, *T. leucostaphylum*, and *T. angustifolium* (with 100% support value) further supports their close phylogenetic relationship within the *Tetrastigma* genus. However, the lower support value (67.4%) between *T. leucostaphylum* and *T. angustifolium* suggests that the evolutionary history of these species may be more complex and could be influenced by gene flow or other factors. Phylogenetic analysis based on the chloroplast genome provides a reliable framework for understanding the genetic relationships among species within the *Tetrastigma* genus. However, the lower support values for some branches indicate that further research is needed to fully resolve the evolutionary history of these species. Future studies could include more molecular markers and expand taxonomic sampling, including more *Tetrastigma* species and related genera, to gain a more comprehensive understanding of their phylogenetic relationships.

#### 3.6.2. Divergence Time Estimation

A time-calibrated tree allows for researchers to better understand the phylogenetic relationships, evolutionary history, and other aspects among species, playing a crucial role when conventional methods are ineffective [41]. This study, through the construction of a Bayesian phylogenetic tree, reveals detailed divergence times for 41 species within the Vitaceae family, providing profound insights into significant evolutionary events within the family. Notably, the topologies of the Bayesian tree and the Maximum Likelihood (ML) tree are generally consistent, with only minor differences at nodes with low bootstrap values. In the Bayesian tree, *T. leucostaphylum* and *T. pachyphyllum* show a closer relationship, unlike *T. angustifolium* in the ML tree. This might reflect the advantages of the Bayesian method in handling evolutionary rate variation and time calibration.

First, the divergence time between the outgroup *Leea guineensis* and the Vitaceae family is approximately 96.93 Mya (95% HPD: 86.53–107.67 Mya), occurring in the Late Cretaceous. This result not only confirms the validity of using *Leea guineensis* as an outgroup prior, but also emphasizes its distant relationship with the Vitaceae family, consistent with previous findings [42,43]. The classification of *Leea* has been debated; some scholars consider *Leea* a part of the Vitaceae family. However, modern perspectives based on morphological traits such as upright shrub habits and the absence of tendrils classify it as a separate family, Leeaceae. The divergence time between *Leea* and the Vitaceae family during the Cretaceous period, significantly earlier than within-family divergences, supports this separation.

Within the Vitaceae family, the Cayratieae subfamily includes the genera *Tetrastigma*, *Pseudocayratia*, *Cayratia*, and *Cyphostemma*. For *Tetrastigma*, results indicate that *Pseudocayratia* and *Tetrastigma* diverged during the Oligocene period. Previous reports mostly place this divergence in the Eocene period [23,24], earlier than our study, but consistent at the species level for specific divergence times, such as *T. rafflesiae* and *T. lawsonii*, diverging around 15 Mya, aligning with previous findings [42]. This suggests significant divergence events in *Tetrastigma* during both the Oligocene and Miocene periods. However, some studies analyzing the divergence time of *Cyphostemma* propose that the divergence of *Tetrastigma* can be traced back to the Cretaceous period [43], much earlier than the Oligocene period. This discrepancy is related to various factors, such as the choice of fossil calibration points, molecular clock models, and the type of molecular data used, such as nuclear or chloroplast gene markers. Empirical data have a limitation in that the actual divergence times are seldom known, making it difficult to judge the best method when age estimates differ [44]. Nonetheless, it is clear that significant events occurred between the Oligocene and Cretaceous periods. A recent study reveals a profound history of extinction and dispersal in the Neotropics, based on fossil seeds of Vitaceae from the Cenozoic, documenting the extinction patterns of the family [45]. Furthermore, this study found that multiple significant divergence events occurred before the Pleistocene. Specifically, three subclades of *Tetrastigma* diverged during the Miocene (19.54 Mya) and the Pliocene (5.14 Mya and 4.61 Mya), indicating that the genus underwent multiple adaptive radiations and evolutionary events throughout its geological history. Outside of the Cayratieae subfamily, the relationship between the genera Nekemias and *Ampelopsis* within the family Vitaceae has been estimated to diverge at a time close to that of the genus *Vitis*, consistent with a previous study [46].

### 3.7. Selective Pressure Analysis

#### 3.7.1. Selective Pressure Analysis of the *Tetrastigma* Genus

Selective pressure, also known as evolutionary pressure, refers to the external forces exerted on an organism during its evolutionary process, thereby altering the direction of this process. Darwin’s concept of natural selection, or survival of the fittest, indicates that selective pressures from the natural environment enable those organisms best adapted to survive and reproduce. The *Ka/Ks* analysis is crucial in studying the molecular evolution of nucleic acids. It represents the ratio between the nonsynonymous substitution rate (*Ka*) and the synonymous substitution rate (*Ks*) of two protein-coding genes. This ratio can determine whether selective pressure is acting on a protein-coding gene.

(1) $$\mathrm{Ka}=\frac{Number\;of\;nonsynonymous\;SNPs}{Number\;of\;nonsynonymous\;sites},$$

(2) $$\mathrm{Ks}=\frac{Number\;of\;synonymous\;SNPs}{Number\;of\;synonymous\;sites}.$$

The *Ka/Ks* ratio reveals the type of selection acting on a gene: *Ka* >> *Ks* or *Ka/Ks* >> 1 indicates positive selection; *Ka* = *Ks* or *Ka/Ks* = 1 indicates neutral evolution; *Ka* << *Ks* or *Ka/Ks* << 1 indicates purifying selection.

The selective pressure analysis for *Tetrastigma angustifolium* and other *Tetrastigma* species suggests that some genes in the *Tetrastigma* genus may play a significant role in adapting to environmental pressures. Purifying selection is widespread, with most genes in the *Tetrastigma* species showing a *Ka/Ks* ratio of less than one, indicating that they are primarily influenced by purifying selection, where natural selection tends to remove harmful non-synonymous mutations to maintain the stability of these genes. The positive selection of the *atpF* gene may be related to its function in photosynthesis, while the positive selection of the *rpl22* gene may be related to the efficiency or accuracy of protein synthesis, which could be downregulated or upregulated under certain stress conditions to help cells adapt to environmental changes. Amanda et al.’s research has confirmed that *rpl22* may be involved in the cell’s response to certain stress conditions, helping cells adapt to environmental changes by regulating its expression levels [47]. The positive selection of the rbcL and *ccsA* genes may be related to the adaptability of these species in specific ecological niches. The *rbcL* gene encodes a key enzyme in photosynthesis, and the *ccsA* gene participates in the biosynthesis of chloroplasts. The positive selection of these genes may indicate that the *Tetrastigma* species have undergone specific adaptive evolution in photosynthesis and chloroplast function. The results of the *Ka/Ks* analysis also correspond with the phylogenetic tree constructed in this study.

#### 3.7.2. Selective Pressure Analysis of Vitaceae Family

This study found that the *rpl32* gene in most Vitaceae species is under significant positive selection, excluding species from the genera *Pseudochayratia*, *Cayratia*, and *Cyphosemma*. Meanwhile, the *rps16* gene exhibited a stable neutral situation, with *Ka/Ks* ratios close to one even in the *Vitis* species. Both *rps16* and *rpl32* encode ribosomal proteins that play critical roles in protein synthesis. A study on rice highlighted the importance of the *rpl32* gene under abiotic stress conditions [48]. Although this study focused on *rpl32* genes located on chromosomes 8 and 9 rather than the chloroplast genome, it suggests potential functions for chloroplast *rpl32* due to gene transfer and exchange involving nuclear, mitochondrial, and chloroplast genes. A study on *Euphorbia schimperi* also revealed the evolutionary fate of *rpl32* and *rps16* [49]. Similarly, research has identified the transfer of the *rpl32* gene from the chloroplast to the nuclear genome, accompanied by the acquisition of new transit peptides [50]. Studies on the Ranunculaceae family reported pseudogenization of the *rpl32* gene, with deletions near the 5′ end leading to internal stop codons [51]. Additionally, chloroplast-encoded *rps16* has been replaced by nuclear-encoded *rps16* products, indicating possible pseudogenization of *rps16* as well [52].

The *Ka/Ks* ratios in the *Vitis* genus are particularly noteworthy, especially for the *rpl33* gene. The *rpl33* gene exhibited exceptionally high *Ka/Ks* ratios, indicating strong positive selection, consistently observed among *Vitis* species sampled in this study. The *rpl33* gene encodes another ribosomal protein, and a study on tobacco demonstrated that *rpl33* is essential for maintaining adequate plastid translation capacity under low-temperature stress [53]. Similarly, in *Arabidopsis*, chloroplast-encoded *rpl33* has been shown to be necessary for survival under cold stress [53]. These findings suggest that *rpl33* may influence plant tolerance to cold stress. This unique feature of *Vitis* aligns with previous divergence time estimates, which suggest that *Vitis* diverged approximately 32.518 million years ago, during the Oligocene period, consistent with previous studies [54]. The reduction in atmospheric CO_2_ levels during the Oligocene likely caused global cooling [55]. The Eocene–Oligocene transition around 34 million years ago is one of the most significant cooling events in Cenozoic climate evolution. Given the high *Ka/Ks* ratio of the *rpl33* gene, it is plausible that mutations in the ancestral *rpl33* gene of *Vitis* enabled its survival during the Oligocene global cooling event. This timing aligns well with other evidence, such as geographic relationships, which require further investigation. However, it is certain that *rpl33* in the genus *Vitis* is a key locus distinguishing it from other genera within the Vitaceae family. In addition to the genera involved in this study, previous research has found that *rpl33* in the genera *Cissus*, *Parthenocissus*, and *Clematicissus* is under significant purifying selection [56,57]. Besides *rpl33*, the *ndhB* gene in *Vitis* also showed significant positive selection, possibly related to the photoperiod [58].

The genera *Ampelopsis* and *Nekemias* showed significantly reduced *Ka/Ks* ratios for the *psaI* gene, all below 0.5, indicating strong purifying selection. However, the study by Zhang et al. suggests that psaI in the genus *Ampelopsis* is under significant positive selection, attributing this to the creeping growth habit of the species, which benefits from enhanced competition for sunlight [57]. The precise function of the *psaI* gene remains unclear, as studies attempting to knock out *psaI* found that PSI redox reactions do not require *psaI* [59]. Unfortunately, the study does not specify the reference species used for selective pressure analysis, leading us to hypothesize that the differences may be due to the choice of reference species. Another similar study, which conducted a comparative analysis between the genera *Vitis* and *Ampelopsis*, concluded that *psaI* is under significant purifying selection, consistent with our findings [60]. For *rpl22*, a study comparing protein-coding genes between *Ampelopsis* and *Vitis* found that the *Ka/Ks* ratio of the *rpl22* gene was significantly greater than one [61], contrary to our findings. Our study found that relative to the Leeaceae family, the *Ka/Ks* value for the *rpl22* gene is generally close to one or zero, with no values significantly greater than one. This discrepancy may be due to pseudogenization of the *rpl22* gene [62] or the choice of study samples.

Using *Leea guineensis* as a reference, this study identified common features among Vitaceae species, such as the significant positive selection of the *rpl32* gene. The high *Ka/Ks* ratio of the *ycf2* gene also distinguishes *Tetrastigma* species. The *Ka/Ks* ratios of *ycf2* in species outside the genus *Tetrastigma* align with the findings of Zhang et al. In the genera *Ampelopsis* and *Nekemias*, the *Ka/Ks* ratios of *ycf2* are all below one, indicating significant purifying selection [57]. Hypothetical chloroplast open reading frames (*ycf*) are sequences with unknown functions in the plastid genome [63], similar to *ycf1*. The high variability of *ycf2* makes it a potential DNA barcode at the species level. However, like *rpl22* and *rps16*, *ycf2* may also undergo pseudogenization [64]. Previous divergence time estimates suggest that *Tetrastigma* diverged earlier than *Vitis*, *Ampelopsis*, and Nekemias, consistent with earlier studies [24]. The variability of *ycf2* may have been beneficial for the evolution of *Tetrastigma* and thus retained. In contrast to *Vitis*, *Tetrastigma* showed significant purifying selection for genes like *ndhB* and *rpl33*.

Notable differences in selective pressure patterns were observed in certain genera, such as *Pseudocayratia*, *Cayratia*, and *Cyphosemma*, particularly in the *rps16*-*rpl32* region. These genera exhibited purifying selection on several genes, including *rps16*, *psaI*, *psaC*, *ndhB*, and *rpl32*. Furthermore, a study calculated the *Ka/Ks* ratios for the genus *Cissus* and, similar to *Pseudochayratia, Cayratia*, and *Cyphosemma*, observed significant purifying selection at loci such as *rps16* and *psaI* [56], contrasting with the positive or neutral selection generally found in other Vitaceae genera. In addition, for the genera *Cissus* and *Cyphostemma*, Zecca et al. identified their distinctiveness in terms of increased substitution rates. Our findings also support those of Zecca et al. [65]. Further research is needed to explore the reasons for these differences, given the limited chloroplast genome data for these species.

## 4. Materials and Methods

### 4.1. Sequence Assembly and Annotation

Genomic sequencing data for 10 *Tetrastigma* species were downloaded from NCBI, and the SRA files were converted into paired-end sequencing files using NCBI’s sra toolkit (v2.11.3). Data quality control of the raw data was performed using Fastp (v0.12.4) [66], resulting in clean_data. The filter_reads.pl script from NOVOplasty (v4.3.1) [67], with the published chloroplast genome of *Tetrastigma hemsleyanum* as the reference sequence, was utilized to filter chloroplast genome reads from the clean_data of the 10 species. Assembly was conducted using Getorganelle (v1.7.7.0) [21], producing circular assemblies for all 10 species that conform to the quadripartite structure of chloroplast genomes. The assembled sequences were used as references for alignment with the corresponding raw data, with a kmer setting of 125, ensuring good coverage of chloroplast genome reads for all ten species and resulting in assemblies without N-base.

Theoretically, there are homologous regions between the chloroplast genome and the nuclear genome. By aligning with reference sequences, homologous regions in the nuclear genome can also be identified. However, these homologous sequences do not affect the assembly process. This is because the copy number of these sequences in the chloroplast genome is significantly higher than in the nuclear genome, resulting in a much higher sequencing depth for the chloroplast genome in the same dataset. Typically, the data used have low sequencing depth for the nuclear genome, which makes it easy for these sequences to be discarded during assembly, thereby directly yielding a complete assembly result. Even when using high-depth data, the depth of the chloroplast genome remains much higher than that of the nuclear genome, facilitating the removal of nuclear genome fragments during assembly.

Chloroplast genome data for an additional five *Tetrastigma* species were downloaded from NCBI. Sequencing data for five species within the *Vitaceae* family were also downloaded, filtered, and assembled for the construction of a phylogenetic tree. GeSeq [68] was used to annotate the chloroplast genomes of 15 *Tetrastigma* species, and annotations were manually refined using the Geneious (R9.0.2) [69]. Physical circular maps of the plant chloroplast genomes were created using the OGDRAW (v1.3.1) [70].

### 4.2. Sequence Analysis and Statistics

Based on the assembly and annotation results, the lengths, GC content, and other basic information of the chloroplast genomes for the 15 *Tetrastigma* species were calculated. The MISA microsatellite finder (v2.1, 25 August 2020) [71] was used to identify Simple Sequence Repeats (SSRs) in the *Tetrastigma* chloroplast genomes. The repeat units included mononucleotide, dinucleotide, trinucleotide, tetranucleotide, pentanucleotide, and hexanucleotide repeats, with minimum repeat parameters set at 10, 5, 4, 3, 3, and 3, respectively. The shared CDS sequences of 15 *Tetrastigma* species were extracted, codon usage and relative synonymous codon usage (RSCU) were analyzed, with RSCU values calculated and visualized.

### 4.3. Genome Comparison and Sequence Divergence Analyses

Information extracted from the annotation files was used for chloroplast genome comparison analysis with mVISTA (v2.0). DnaSP V6 [72] was utilized to calculate the nucleotide diversity (Pi) values for the 15 species, with a sliding window of 600 bp and a step size of 200 bp. The results were visualized using Excel, and the genes corresponding to the diversity hotspots were annotated. IRScope (16 March 2018) [73] was employed to illustrate the contraction and expansion of the IR/SC region boundaries.

### 4.4. Phylogenetic Analysis

The raw sequencing data for five species within the Vitaceae family were downloaded from NCBI, and their corresponding chloroplast genomes were assembled. MAFFT (v7.310) [74] was used to perform multiple sequence alignment of the chloroplast genomes for 20 species. The aligned sequences were then imported into MEGA11 [75] to determine the best DNA/protein model (ML). Using the Maximum Likelihood method, the GTR+G+I model was selected (Table S3), with the Bootstrap set to 1000, to construct the phylogenetic tree.

### 4.5. Divergence Time Estimation

A total 41 species were used for analysis, including those from the genera *Ampelopsis, Vitis*, and *Tetrastigma* in the Vitaceae family, along with an outgroup reference from the genus *Leea*. The common CDS sequences of the 41 species were extracted and aligned using MAFFT (v7.310), resulting in an alignment length of 64,116 bp. BEAST (v2.7.7) was used to estimate the divergence time of the *Tetrastigma* genus. Phylosuite (v1.2.3) was employed to calculate the best model, and the TVM+F+G4 model was chosen as the substitution model. The Gamma Category Count was set to 4, Shape to 1.1, and Frequencies to Empirical. Strict molecular clock was used. The prior tree was determined to be the Yule model, with four calibration points (a–d) set to normal distributions: (a) the deep calibration point for *Leea* and Vitaceae, with a mean of 85 Mya and a SD of 4 Mya, providing a 95% confidence interval (CI) of 77.6–92.6 Mya [76]; (b) based on the existing fossil record of *Tetrastigma* (https://paleobiodb.org/classic/checkTaxonInfo?taxon_no=418545&is_real_user=1, accessed on 12 July 2024.), with a mean of 61 Mya and a SD of 2.55 Mya, providing a 95% CI of 56.0–66.0 Mya; (c) for the genus *Ampelopsis*, with a mean of 41.2 Mya and a SD of 9.59 Mya, providing a 95% CI of 23.4–61.0 Mya [23]; (d) for the subgenus *Vitis*, with a mean of 3.2 Mya and a SD of 0.5 Mya, providing a 95% CI of 2.22–4.18 Mya [77].

We performed two runs, each consisting of 50,000,000 generations, and used Tracer to check for convergence. The results of the runs were combined using LogCombiner (v2.7.7). Finally, the resulting tree was obtained using TreeAnnotator (v2.7.7) with 20% burn-in.

### 4.6. Selective Pressure Analysis

Coding sequences (CDSs) and protein sequences were extracted from the GenBank files of 15 *Tetrastigma* chloroplast genomes. BLASTN (v2.14.0+) was utilized to compare other protein sequences against a reference protein sequence to identify the best matches, thereby obtaining homologous protein sequences. MAFFT (v7.310) [75] was employed for automatic alignment of the homologous protein sequences. The aligned protein sequences were mapped back to the coding sequences to obtain aligned CDS. The *Ka* and *Ks* values were computed using KaKs_Calculator3 [78] based on the MLWL method.

Python (v3.9.6) was used to analyze *Ka/Ks* values for multiple genes from Excel files. The pandas library (v2.2.2) was utilized to read the data. For statistical analysis, a normality test on the *Ka/Ks* values of each gene was conducted using the Shapiro–Wilk test, implemented in the scipy.stats.shapiro (v1.9.1) function. If the *p*-value was greater than the significance level (α = 0.05), the data were considered normally distributed. Subsequently, hypothesis testing was performed. For normally distributed data, a one-sample *t*-test (scipy.stats.ttest_1samp) was used to determine whether the mean *Ka/Ks* value significantly differed from one. For non-normally distributed data, the Mann–Whitney U test (scipy.stats.mannwhitneyu) was applied to assess whether the *Ka/Ks* values significantly differed from one. For confidence interval estimation, the t-distribution (scipy.stats.t.ppf) was used to calculate the 95% confidence interval for normally distributed data. For non-normally distributed data, the interquartile range (25th and 75th percentiles) was used as a non-parametric confidence interval. In the interpretation of results, significance was determined if the *p*-value was less than the significance level (α = 0.05). If the *t*-test statistic was greater than zero and the result was significant, or if the Mann–Whitney U test indicated that the mean *Ka/Ks* value was greater than one and the result was significant, positive selective pressure was inferred. Conversely, if the *t*-test statistic was less than zero and the result was significant, or if the Mann–Whitney U test indicated that the mean *Ka/Ks* value was less than one and the result was significant, purifying selective pressure was inferred. Otherwise, no significant selective pressure was concluded.

## 5. Conclusions

This study assembled the complete chloroplast genomes of ten species within the genus *Tetrastigma* and annotated and analyzed the chloroplast genomes of fifteen species within the genus. The results indicate that: (1) through SSR analysis, we detected a total of 1139 SSRs in the chloroplast genomes, with a higher proportion of A/T repeats, and identified unique pentanucleotide repeats in five plants, which have potential as SSR markers. Comparative analysis identified several polymorphic hotspots in the chloroplast genomes of *Tetrastigma* species (*rps16-trnR^UCU^*, *trnC^GCA^-trnT^GGU^*, *trnS^UGA^-trnG^GCC^*, and *ndhF-trnL^UAG^*; 10 in LSC: *rps16*, *rps16-trnQ*, *trnS*, *trnD*, *psbC-trnS-psbZ*, *accD-psaI*, *psbE-petL-petG*, *clpPI*, *rpl16*, *rps3-rps19*; 2 in SSC: *ndhF-ndhD and ycf1*). These loci and the detected SSRs can be used to develop DNA markers. (2) Phylogenetic analysis provided a high-confidence understanding of the evolutionary relationships within the *Tetrastigma* genus. Phylogenetic analysis based on the entire chloroplast genome grouped *Tetrastigma* species into a monophyletic clade, with *T. serrulatum*, *T. annamense*, *T. thorsborneorum*, and *T. pyriforme* showing close relationships. (3) We estimated the divergence times based on 40 Vitaceae species. The differentiation of the Vitaceae family can be traced back to 96.96 Mya, while *Tetrastigma* species can be traced back to the Oligocene, with diversification occurring during the Miocene and Pliocene, and possibly divergence events before the Eocene. This timeline is crucial for comparing the evolutionary history of *Tetrastigma* with other genera in the Vitaceae family. Additionally, this study used a strict molecular clock for analysis, showing good convergence; the evaluation of other molecular clocks requires further study. (4) This study investigated the selective pressures experienced by key genes in the *Tetrastigma* genus, finding significant positive selection in genes such as *atpF*, *rbcL*, and *accD*. Furthermore, at the broader Vitaceae family level, selective pressure analysis highlighted evolutionary features distinguishing the *Tetrastigma* genus from other genera, with most genes under purifying selection. The high *Ka/Ks* ratio of the *rpl32* gene is a notable feature of Vitaceae evolution. Additionally, significant positive selection in the *rpl33* and *ndhB* genes played an important role in the evolution of the *Vitis* genus, while significant positive selection in the *ycf2* gene is a characteristic of *Tetrastigma* evolution. This part of the study helps to understand how the *Tetrastigma* genus adapts to its environment and how these evolutionary pressures compare to those in other genera of the Vitaceae family. Furthermore, the analysis of the *rpl22* gene’s results differed from previous studies; additionally, this study did not explore the unique selective pressures in the genera *Pseudocayratia, Cayratia*, and *Cyphosemma*, which are worth further research. The results of this study provide valuable data on SSR and SNP markers, phylogenetic relationships, divergence times, and selective pressures. These findings enhance our understanding of the genetic diversity, evolutionary history, and adaptive mechanisms of the *Tetrastigma* genus, laying a solid foundation for future research in population genetics, species identification, and conservation biology of this genus.

## Figures and Tables

**Table 1:** Summary of 15 *Tetrastigma* chloroplast genome characteristics.

Species	Genome Size	LSC Length	IR Length	SSC Length	Total GC Content	GC Content of LSC	GC Content of IR	GC Content of SSC	Number of Total Genes	Number of CDS	Number of rRNA Genes	Number of tRNA Genes
*T. angustifolium* (NC_029339)	159,988	88,107	26,510	18,861	37.55%	35.54%	42.89%	31.77%	131	86	8	37
*T. annamense*	160,736	88,821	26,288	19,339	37.56%	35.35%	42.98%	31.55%	132	87	8	37
*T. canarense*	160,312	88,010	26,627	19,048	37.57%	35.53%	42.82%	31.77%	132	87	8	37
*T. cauliflorum*	160,382	88,238	26,513	19,118	37.62%	35.50%	42.87%	31.65%	131	86	8	37
*T. hemsleyanum*	159,889	87,901	26,511	18,966	37.53%	35.58%	42.89%	31.77%	132	87	8	37
*T. lawsonii* (NC_061673)	160,360	88,117	26,469	19,306	37.53%	35.50%	42.93%	31.57%	131	86	8	37
*T. leucostaphylum*	159,387	87,499	26,506	18,876	37.43%	35.59%	42.88%	31.71%	131	86	8	37
*T. nilagiricum*	160,116	88,021	26,492	19,111	37.58%	35.63%	42.90%	31.69%	132	87	8	37
*T. pachyphyllum*	159,317	87,384	26,529	188,875	37.49%	35.62%	42.86%	31.79%	131	86	8	37
*T. planicaule* (NC_057118)	160,323	88,181	26,523	19,096	37.39%	35.52%	42.87%	31.66%	131	86	8	37
*T. pyriforme*	160,893	88,979	26,319	19,276	37.48%	35.35%	42.97%	31.51%	131	86	8	37
*T. rafflesiae* (NC_061671)	159,805	87,920	26,306	19,273	37.55%	35.63%	42.99%	31.62%	131	86	8	37
*T. serrulatum*	160,113	88,084	26,441	19,147	37.50%	35.34%	42.85%	31.41%	132	87	8	37
*T. thorsborneorum*	160,676	88,159	26,934	18,649	37.48%	35.43%	42.73%	31.62%	131	86	8	37
*T.voinierianum* (NC_061711)	160,005	87,874	26,510	19,111	37.35%	35.59%	42.87%	31.65%	131	86	8	37

**Table 2:** Gene types of *Tetrastigma* chloroplast genome.

Category	Gene Group	Gene Name
Photosynthesis	Subunits of photosystem I	*psaA*, *psaB*, *psaC*, *psaI*, *psaJ*
	Subunits of photosystem II	*psbA*, *psbB*, *psbC*, *psbD*, *psbE*, *psbF*, *psbH*, *psbI*, *psbJ*, *psbK*, *psbL*, *psbM*, *psbT*, *psbZ*
	Subunits of NADH dehydrogenase	ndhA *, ndhB *(2), *ndhC*, *ndhD*, *ndhE*, *ndhF*, *ndhG*, *ndhH*, *ndhI*, *ndhJ*, *ndhK*
	Subunits of cytochrome b/f complex	*petA*, petB *, petD *, *petG*, *petL*, *petN*
	Subunits of ATP synthase	*atpA*, *atpB*, *atpE*, atpF *, *atpH*, *atpI*
	Large subunit of rubisco	*rbcL*
Self-replication	Proteins of large ribosomal subunit	*rpl14*, rpl16 *, rpl2 *(2), *rpl20*, *rpl22*, *rpl23(2)*, *rpl32*, *rpl33*, *rpl36*
	Proteins of small ribosomal subunit	*rps11*, rps12 **(2), *rps14*, *rps15*, rps16 *, *rps18*, *rps19*, *rps2*, *rps3*, *rps4*, *rps7(2)*, *rps8*, *ψrps19-fragment*
	Subunits of RNA polymerase	*rpoA*, *rpoB*, rpoC1 *, *rpoC2*
	Ribosomal RNAs	*rrn16(2)*, *rrn23S(2)*, *rrn4.5(2)*, *rrn5(2)*
	Transfer RNAs	trnA^UGC^ *(2), *trnC^GCA^*, *trnD^GUC^*, *trnE^UUC^*, *trnF^GAA^*, *trnG^GCC^*, *trnH^GUG^*, *trnI^CAU^(2)*, trnI^GAU^ *(2), trnK^UUU^ *, *trnL^CAA^(2)*, trnL^UAA^ *, *trnL^UAG^*, *trnM^CAU^*, *trnN^GUU^(2)*, *trnP^UGG^*, *trnQ^UUG^*, *trnR^ACG^(2)*, *trnR^UCU^*, trnS^CGA^ *, *trnS^GCU^*, *trnS^GGA^*, *trnS^UGA^*, *trnT^GGU^*, *trnT^UGU^*, *trnV^GAC^(2)*, trnV^UAC^ *, *trnW^CCA^*, *trnY^GUA^*, *trnfM^CAU^*
Other genes	Maturase	*matK*
	Protease	clpP1 **
	Envelope membrane protein	*cemA*
	Acetyl-CoA carboxylase	*accD*
	c-type cytochrome synthesis gene	*ccsA*
	Translation initiation factor	*infA*
	Other	pafI **, *pafII*, *pbf1*
Genes of unknown function	Conserved hypothetical chloroplast ORF	*ycf1*, *ψycf1*, *ycf2(2)*

Notes: Gene *: Gene with one intron; Gene **: Gene with two introns; ψGene: Pseudo gene; *Gene(2)*: Number of copies of multi-copy genes.

**Table 3:** SSRs detected in the *Tetrastigma* chloroplast genome P1-mononucleotide, P2-dinucleotide, P3-trinucleotide, P4-tetranucleotide, P5-pentanucleotide.

Species	P1	P2	P3	P4	P5	Total	LSC	IRb	SSC	IRa
*T. angustifolium*	55	13	2	7	0	77	54	3	17	3
*T. annamense*	47	17	3	4	2	73	59	1	12	1
*T. canarense*	56	15	1	5	0	77	58	2	15	2
*T. cauliflorum*	61	13	2	7	0	83	59	3	18	3
*T. hemsleyanum*	53	11	2	4	0	70	49	2	17	2
*T. lawsonii*	64	12	1	6	0	83	56	3	21	3
*T. leucostaphylum*	56	13	2	7	0	78	56	3	16	3
*T. nilagiricum*	57	12	2	7	0	78	54	3	18	3
*T. pachyphyllum*	56	14	2	7	1	80	58	3	16	3
*T. planicaule*	56	14	2	7	0	79	54	3	19	3
*T. pyriforme*	46	16	4	6	1	73	55	2	14	2
*T. rafflesiae*	56	11	2	5	0	74	51	2	19	2
*T. serrulatum*	51	13	1	9	1	75	57	1	16	1
*T. thorsborneorum*	36	18	2	8	0	64	47	1	15	1
*T. voinierianum*	55	12	2	6	0	75	48	3	21	3

**Table 4:** CDS with significant positive selection among the four genera.

Genus	CDS	*Ka/Ks* Mean	Selection	*p*-Value	Significant	Normality	Test
*Ampelopsis*	*ndhB*	1.201224	positive selection	0.006501702	TRUE	FALSE	Mann–Whitney U
	*rpl32*	2.30669	positive selection	0	TRUE	TRUE	*t*-test
Nekemias	*rpl32*	1.38791	positive selection	0	TRUE	TRUE	*t*-test
*Tetrastigma*	*rpl32*	1.125571	positive selection	9.51019 × 10^−6^	TRUE	FALSE	Mann–Whitney U
	*ycf2*	1.260423	positive selection	4.58275 × 10^−6^	TRUE	TRUE	*t*-test
*Vitis*	*ndhB*	1.637811	positive selection	0.000122063	TRUE	FALSE	Mann–Whitney U
	*rpl32*	1.808506	positive selection	7.03107 × 10^−5^	TRUE	FALSE	Mann–Whitney U
	*rpl33*	4.061119	positive selection	0.00206442	TRUE	FALSE	Mann–Whitney U

## Data Availability

All raw data from this study can be downloaded from NCBI, with the corresponding accession numbers listed in the Supplementary Materials (Table S1). Additionally, data generated during the analysis can be obtained by contacting the corresponding author.

## Supplementary Materials

The following supporting information can be downloaded at: https://www.mdpi.com/article/10.3390/ijms25158290/s1.
