# Supplementary material for: Decoding the Chloroplast Genome of Tetrastigma (Vitaceae): Variations and Phylogenetic Selection Insights
Source: Int J Mol Sci. 2024 Jul 29;25(15):8290. doi: 10.3390/ijms25158290 (PMC11312916; doi:10.3390/ijms25158290)
Supplement: Supplementary file 1 [file ijms-25-08290-s001.zip › ijms-3051370-supplementary.pdf]

Table S1: The chloroplast genome appeared in the study.and its corresponding NCBI Accession number and SRA number

Species	Accession number	SRA number
Tetrastigma hemsleyanum	NC_029339	/
Tetrastigma lawsonii	NC_061673	/
Tetrastigma planicaule	NC_057118	/
Tetrastigma rafflesiae	NC_061671	/
Tetrastigma voinierianum	NC_061711	/
*Tetrastigma angustifolium**	PP657251	SRR25608221
*Tetrastigma annamense**	PP657252	SRR25608213
*Tetrastigma canarense**	PP657253	SRR25608222
*Tetrastigma cauliflorum**	PP657254	SRR25608211
*Tetrastigma leucostaphylum**	PP657255	SRR25608202
*Tetrastigma nilagiricum**	PP657256	SRR25608228
*Tetrastigma pachyphyllum**	PP657257	SRR25608217
*Tetrastigma pyriforme**	PP657258	SRR25608210
*Tetrastigma serrulatum**	PP657259	SRR25608226
*Tetrastigma thorsborneorum**	PP657260	SRR25608231

Notes: This study assembled chloroplast genomes of species with ' *'.

Table S2: Statistics of repeat types classified by repeat unit type.

Species / Type	*T.angustifolium*	*T.annamense*	*T.canarense*	*T.cauliflorum*	*T.hemsleyanum*	*T.lawsonii*	*T.leucostaphylum*	*T.nilagiricum*	*T.pachyphyllum*	*T.planicaule*	*T.pyriforme*	*T.rafflesiae*	*T.serrulatum*	*T.thorsborneorum*	*T.voinierianum*	**Total**	**Proportion**
A/T	54	45	55	60	53	62	55	55	55	56	43	54	51	36	55	789	69.27%
C/G	1	2	1	1	0	2	1	2	1	0	3	2	0	0	0	16	1.40%
AG/CT	1	1	1	1	1	1	1	1	1	1	1	1	1	1	1	15	1.32%
AT/AT	12	16	14	12	10	11	12	11	13	13	15	10	12	17	11	189	16.59%
AAT/ATT	2	2	1	2	2	1	2	2	2	2	3	2	1	2	2	28	2.46%
AGC/CTG	0	1	0	0	0	0	0	0	0	0	1	0	0	0	0	2	0.18%
AAAG/CTTT	1	0	1	1	0	1	1	1	1	1	1	2	1	2	1	15	1.32%
AAAT/ATTT	3	2	2	3	2	3	3	3	3	3	4	2	7	3	2	45	3.95%
AAGG/CCTT	1	0	0	1	0	0	1	1	1	0	0	0	0	0	0	5	0.44%
AATC/ATTG	1	1	1	1	1	1	1	1	1	1	1	1	1	1	1	15	1.32%
AATT/AATT	1	1	0	1	1	1	1	1	1	2	0	0	0	2	2	14	1.23%
ACAT/ATGT	0	0	1	0	0	0	0	0	0	0	0	0	0	0	0	1	0.09%
AACAT/ATGTT	0	0	0	0	0	0	0	0	0	0	1	0	0	0	0	1	0.09%
AAGAT/ATCTT	0	1	0	0	0	0	0	0	0	0	0	0	0	0	0	1	0.09%
AATAG/ATTCT	0	1	0	0	0	0	0	0	0	0	0	0	0	0	0	1	0.09%
ATATC/ATATG	0	0	0	0	0	0	0	0	1	0	0	0	0	0	0	1	0.09%
AGAGAT/ATCTCT	0	0	0	0	0	0	0	0	0	0	0	0	1	0	0	1	0.09%

total	77	73	77	83	70	83	78	78	80	79	73	74	75	64	75	11<br>39

Table S3: Maximum Likelihood fits of 24 different nucleotide substitution models.

Model	Parameters	BIC	AICc	*lnL*	(+*I*)	(+*G*)	*R*	*f*(A)	*f*(T)	*f*(C)	*f*(G)	*r*(AT)	*r*(AC)	*r*(AG)	*r*(TA)	*r*(TC)	*r*(TG)	*r*(CA)	*r*(CT)	*r*(CG)	*r*(GA)	*r*(GT)	*r*(GC)
GTR+G+I	47	652521.978	651912.033	-325909.016	0.65	0.84	0.96	0.309	0.316	0.191	0.184	0.039	0.065	0.091	0.038	0.094	0.066	0.106	0.156	0.039	0.153	0.113	0.040
GTR+G	46	652782.676	652185.708	-326046.853	n/a	0.09	0.95	0.309	0.316	0.191	0.184	0.040	0.065	0.091	0.039	0.094	0.065	0.106	0.155	0.039	0.153	0.112	0.040
HKY+G+I	43	653782.128	653224.092	-326569.046	0.65	0.85	1.09	0.309	0.316	0.191	0.184	0.073	0.044	0.099	0.071	0.103	0.043	0.071	0.170	0.043	0.166	0.073	0.044
T92+G+I	41	653785.658	653253.577	-326585.788	0.65	0.85	1.09	0.312	0.312	0.188	0.188	0.072	0.043	0.101	0.072	0.101	0.043	0.072	0.168	0.043	0.168	0.072	0.043
TN93+G+I	44	653796.825	653225.812	-326568.905	0.65	0.85	1.09	0.309	0.316	0.191	0.184	0.073	0.044	0.099	0.071	0.103	0.043	0.071	0.170	0.043	0.166	0.073	0.044
HKY+G	42	653997.077	653452.019	-326684.009	n/a	0.09	1.04	0.309	0.316	0.191	0.184	0.075	0.045	0.097	0.073	0.100	0.044	0.073	0.166	0.044	0.162	0.075	0.045
T92+G	40	654000.722	653481.619	-326700.809	n/a	0.09	1.04	0.312	0.312	0.188	0.188	0.074	0.045	0.099	0.074	0.099	0.045	0.074	0.164	0.045	0.164	0.074	0.045
TN93+G	43	654004.386	653446.351	-326680.175	n/a	0.09	1.01	0.309	0.316	0.191	0.184	0.076	0.046	0.092	0.074	0.102	0.044	0.074	0.169	0.044	0.155	0.076	0.046
GTR+I	46	654792.842	654195.874	-327051.936	0.47	n/a	0.94	0.309	0.316	0.191	0.184	0.041	0.065	0.090	0.040	0.094	0.065	0.105	0.155	0.040	0.152	0.112	0.041
HKY+I	42	655969.972	655424.914	-327670.456	0.47	n/a	0.94	0.309	0.316	0.191	0.184	0.079	0.048	0.092	0.077	0.096	0.046	0.077	0.158	0.046	0.155	0.079	0.048
T92+I	40	655973.763	655454.660	-327687.329	0.47	n/a	0.94	0.312	0.312	0.188	0.188	0.078	0.047	0.094	0.078	0.094	0.047	0.078	0.156	0.047	0.156	0.078	0.047
TN93+I	43	655984.939	655426.903	-327670.451	0.47	n/a	0.94	0.309	0.316	0.191	0.184	0.079	0.048	0.092	0.077	0.096	0.046	0.077	0.158	0.046	0.155	0.079	0.048
GTR	45	657065.347	656481.357	-328195.678	n/a	n/a	0.94	0.309	0.316	0.191	0.184	0.042	0.065	0.090	0.041	0.093	0.065	0.105	0.155	0.040	0.151	0.112	0.041
HKY	41	658230.574	657698.493	-328808.246	n/a	n/a	0.94	0.309	0.316	0.191	0.184	0.079	0.048	0.092	0.077	0.096	0.046	0.077	0.158	0.046	0.154	0.079	0.048

T92	39	658234.306	657728.181	-328825.090	n/a	n/a	0.94	0.312	0.312	0.188	0.188	0.078	0.047	0.094	0.078	0.094	0.047	0.078	0.156	0.047	0.156	0.078	0.047
TN93	42	658245.546	657700.488	-328808.243	n/a	n/a	0.94	0.309	0.316	0.191	0.184	0.079	0.048	0.092	0.077	0.095	0.046	0.077	0.158	0.046	0.154	0.079	0.048
K2+G+I	40	665395.641	664876.538	-332398.268	0.66	0.85	1.17	0.250	0.250	0.250	0.250	0.058	0.058	0.135	0.058	0.135	0.058	0.058	0.135	0.058	0.135	0.058	0.058
K2+G	39	665597.795	665091.670	-332506.834	n/a	0.07	1.11	0.250	0.250	0.250	0.250	0.059	0.059	0.131	0.059	0.131	0.059	0.059	0.131	0.059	0.131	0.059	0.059
JC+G+I	39	666578.175	666072.050	-332997.024	0.65	0.85	0.50	0.250	0.250	0.250	0.250	0.083	0.083	0.083	0.083	0.083	0.083	0.083	0.083	0.083	0.083	0.083	0.083
JC+G	38	666834.141	666340.993	-333132.496	n/a	0.08	0.50	0.250	0.250	0.250	0.250	0.083	0.083	0.083	0.083	0.083	0.083	0.083	0.083	0.083	0.083	0.083	0.083
K2+I	39	667629.961	667123.836	-333522.917	0.47	n/a	1.05	0.250	0.250	0.250	0.250	0.061	0.061	0.128	0.061	0.128	0.061	0.061	0.128	0.061	0.128	0.061	0.061
JC+I	38	668892.099	668398.951	-334161.475	0.47	n/a	0.50	0.250	0.250	0.250	0.250	0.083	0.083	0.083	0.083	0.083	0.083	0.083	0.083	0.083	0.083	0.083	0.083
K2	38	669904.548	669411.401	-334667.700	n/a	n/a	1.04	0.250	0.250	0.250	0.250	0.061	0.061	0.128	0.061	0.128	0.061	0.061	0.128	0.061	0.128	0.061	0.061
JC	37	671165.037	670684.866	-335305.433	n/a	n/a	0.50	0.250	0.250	0.250	0.250	0.083	0.083	0.083	0.083	0.083	0.083	0.083	0.083	0.083	0.083	0.083	0.083

Notes: Models with the lowest BIC scores (Bayesian Information Criterion) are considered to describe the substitution pattern the best.
